# Post-acute Myocardial Infarction Complication: A Ventricular Septal Defect

**DOI:** 10.7759/cureus.97216

**Published:** 2025-11-19

**Authors:** Bruna Rodrigues Barbosa, Joana Cartucho, Tatiana Duarte, Ana Paula Pona, Filipe Seixo

**Affiliations:** 1 Internal Medicine, Unidade Local de Saúde do Arco Ribeirinho-Centro Hospitalar Barreiro Montijo, Barreiro, PRT; 2 Cardiology, Unidade Local de Saúde da Arrábida, Setúbal, PRT

**Keywords:** acute myocardial infarction, intra-aortic balloon pump, multidisciplinary approach, percutaneous coronary intervention, ventricular septal defect (vsd)

## Abstract

A ventricular septal defect (VSD) is usually a congenital cardiac condition. In the elderly, although it may be present from birth, VSD can be acquired and manifest as a complication of acute myocardial infarction (AMI), as endocarditis, or as a result of cardiac procedures. It can lead to heart failure and other severe consequences demanding medical intervention.

This case study describes an 80-year-old obese woman who was diagnosed with inferolateral myocardial infarction with right ventricular (RV) involvement after being admitted with symptoms of acute coronary syndrome (ACS). VSD, a rare mechanical complication, was identified as a contributing factor to hemodynamic instability. The patient underwent percutaneous coronary intervention (PCI) to revascularize the occluded right coronary artery (RCA). Hemodynamic support with norepinephrine and levosimendan, along with mechanical ventilation, was crucial for stabilization. Transfer to a tertiary care facility with cutting-edge surgical skills was essential.

This example emphasizes how challenging it can be to manage AMI in elderly people, particularly when unusual side effects like VSD occur. It also highlights how important a multidisciplinary approach is to improving outcomes and optimizing care.

## Introduction

The management of acute coronary syndrome (ACS) in the elderly has been extensively studied. In older populations, when concomitant conditions like diabetes and hypertension are more common, obesity is frequently overlooked despite being a known cardiovascular risk factor. However, recent evidence suggests that obesity may worsen the prognosis of acute myocardial infarction (AMI) by increasing the risk of complications such as ventricular dysfunction and heart failure [1].

Reducing the time to revascularization is crucial in lowering both mortality and complications after an AMI. Acting quickly and restoring blood flow early are linked to better outcomes, particularly in high-risk groups such as the elderly or patients with multiple comorbidities [2,3].

Mechanical complications after an AMI, such as ventricular septal defect (VSD), free wall rupture, and papillary muscle rupture, are rare but can be life-threatening, affecting less than 1% of patients in the current reperfusion era. Ischemic VSD typically develops within 3-5 days after the infarction, although it can sometimes occur even earlier, which may have important clinical consequences. Being aware of this timeline is crucial, as early recognition and timely management can have a major impact on patient outcomes. These uncommon complications further complicate the care of elderly patients with AMI. Rapid identification and prompt surgical intervention are essential to improve outcomes, although treatment is often difficult due to frailty and the presence of multi-organ dysfunction [4-7].

A multidisciplinary team comprising cardiologists, intensivists, and cardiothoracic surgeons is necessary to provide tailored, integrated care to address these concerns [8]. This case focuses on an elderly woman who was admitted to the emergency department with symptoms of ACS and had obesity as her only cardiovascular risk factor. The case illustrates the clinical difficulties involved in these presentations and highlights an uncommon etiology of VSD and the need for detecting it in order to guarantee appropriate therapy and long-term clinical stability.

## Case presentation

An 80-year-old obese woman with no prior known medical history arrived with chest and epigastric pain, sweating, nausea, and a general feeling of unwellness. Before being admitted, she had suffered from malaise and vertigo for three days. She denied taking any medications on a regular basis. Her initial systolic blood pressure (SBP), which was approximately 100 mmHg, improved to 130 mmHg following fluid resuscitation. She was transported by the Medical Emergency and Resuscitation Vehicle.

Upon admission, the patient presented with a Glasgow Coma Scale (GCS) score of 15, a blood pressure of 113/64 mmHg, and a heart rate of 102 bpm. She was calm and oriented, but sweating. Moreover, she was eupneic in ambient air, without signs of respiratory difficulty. Auscultation shows normal lung sounds.

**Figure 1:**
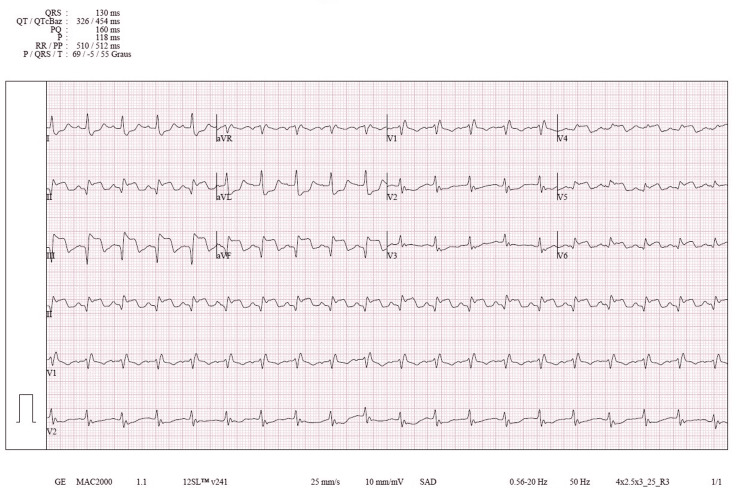
ECG. QRS: QRS duration (duration of the ventricular depolarization complex); QT: QT interval (ventricular depolarization+repolarization); QTcBaz: Bazett-corrected QT interval (QTc calculated using the Bazett formula); PQ: PQ interval/PR interval (time from the onset of atrial depolarization to the onset of ventricular depolarization); P: P wave duration (duration of atrial depolarization); RR: RR interval (time between R waves (heartbeat interval)); PP: PP interval (time between P waves (atrial cycle interval)); P: P-axis (electrical axis of the atria); QRS: QRS-axis (electrical axis of ventricular depolarization); T: T-axis (electrical axis of ventricular repolarization); GE: general electric (manufacturer of the ECG machine); SAD: standard automatic display; ECG: electrocardiogram; aVR: augmented vector right; aVF: augmented vector foot; aVL: augmented vector left

An inferolateral AMI with right ventricular (RV) involvement was supported by the electrocardiogram's (ECG) sinus rhythm, ST-segment elevation in leads II, III, augmented vector foot (aVF), and V3-V6, and ST depression in leads I and augmented vector left (aVL) (Figure 1). Venous blood collections and arterial blood gas analysis were performed, revealing elevated troponin I (>50,000 pg/mL) and metabolic acidosis.

Transthoracic echocardiography (TTE) revealed a non-dilated aortic root and ascending aorta, a mildly thickened but functionally normal aortic valve, a preserved left ventricular ejection fraction (>55%), and akinesis of the inferior septum and inferior wall. The RV was severely dilated with significant systolic dysfunction. Mild mitral regurgitation and mild left atrial dilation were also noted.

**Figure 2:**
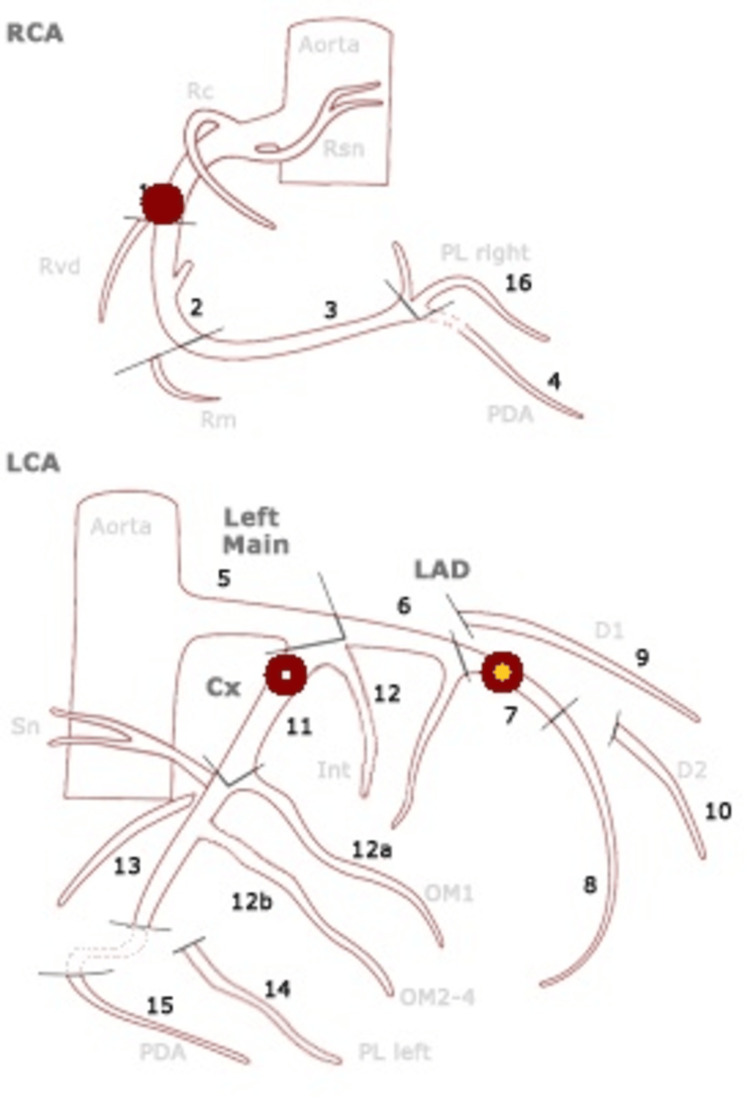
Coronary angiography. RCA: total occlusion (type C, TIMI 0, diffuse ≥20 mm). Cx: 71-90% stenosis (type B2, TIMI 3). LAD artery: 51-70% stenosis (type B1, TIMI 3). Rc: right coronary; Rsn: right sinus; Rvd: right ventricular branch; PL right: posterolateral branch (right); Rm: right marginal branch; Sn: sinus node branch; Int: intermediate branch; D1: diagonal 1; D2: diagonal 2; OM1: obtuse marginal branch 1; OM2-4: obtuse marginal branches 2-4, PL left: posterolateral branch (left); RCA: right coronary artery; TIMI: Thrombolysis in Myocardial Infarction; Cx: circumflex artery; LAD: left anterior descending; LCA: left coronary artery; PDA: patent ductus arteriosus

**Figure 3:**
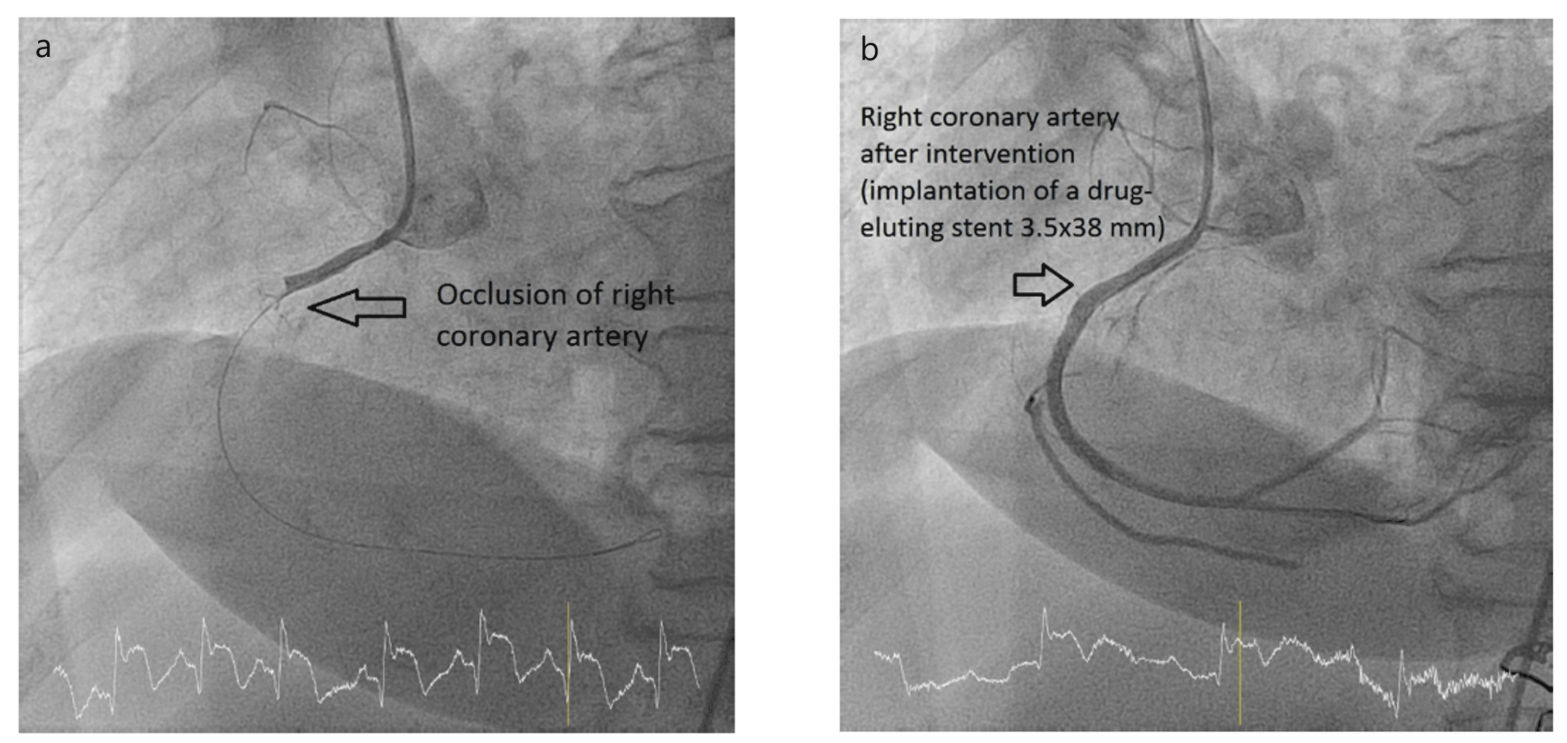
(a) Occlusion of RCA. (b) After intervention. RCA: right coronary artery

Coronary angiography showed right coronary dominance with no significant lesions in the left main stem. A 50-70% lesion was observed in the mid-segment of the left anterior descending artery (LAD) and a 70-90% lesion in the ostial circumflex artery (LCx) (Figure 2). The right coronary artery (RCA) was proximally occluded (Figure 3a) and was treated with percutaneous coronary intervention (PCI) and drug-eluting stent placement (Figure 3b). Despite this, the final flow remained sluggish due to a high thrombotic burden, Thrombolysis in Myocardial Infarction (TIMI) 2.

The patient experienced severe hypotension (SBP 70-80 mmHg) during PCI, necessitating norepinephrine. Arterial blood gas analysis showed a pH of 7.39, a lactate level of 11.5 mmol/L, and a bicarbonate (HCO₃⁻) level of 7.3 mmol/L. Laboratory findings were notable for an elevation of inflammatory markers, the deterioration of renal and hepatic function, and a marked increase in troponin I levels (Table 1). 

**Table 1:** Summary of the patient's blood test results on admission. The bold data fall outside the reference ranges.

Laboratory tests	Patient value	Reference range
Hemoglobin (g/dL)	10.4	12.0-15.6
White blood cell count (×10⁹/L)	14.5	3.9-10.2
Neutrophils (%)	78	40-75
Platelets (×10⁹/L)	155	150-400
International normalized ratio	2.2	0.8-1.2
Glucose (mg/dL)	90	60-109
Urea (mg/dL)	135	7.9-20.9
Creatinine (mg/dL)	2.61	0.55-1.02
Sodium (mmol/L)	131	136-146
Potassium (mmol/L)	4.3	4.0-5.1
Chloride (mmol/L)	104	101-109
Aspartate aminotransferase (U/L)	1782	<31
Alanine aminotransferase (U/L)	2210	<34
Troponin I (pg/mL)	>50000	<16
C-reactive protein (mg/dL)	18.1	<0.5

In the coronary care unit (CCU), the patient was agitated, with cold extremities and a capillary refill time of less than two seconds. She remained in persistent shock after PCI, and a TTE was ordered to evaluate the etiology of shock. Her mean arterial pressure (MAP) was approximately 60 mmHg. Repeat TTE revealed a VSD in the inferior septum, contributing to hemodynamic instability, and confirmed RV dysfunction (Video 1 and Video 2).

**Video 1:** TTE (in the CCU), with Doppler. Identification of VSD in the inferior septum. TTE: transthoracic echocardiography; CCU: coronary care unit; VSD: ventricular septal defect

**Video 2:** TTE (in the CCU). Identification of VSD in the inferior septum. TTE: transthoracic echocardiography; CCU: coronary care unit; VSD: ventricular septal defect

Non-invasive ventilation (NIV) was initiated, followed by intubation and mechanical ventilation (using midazolam, etomidate, and rocuronium) due to progressive respiratory exhaustion. Levosimendan was started, and noradrenaline was continued. Intra-aortic balloon pump placement was considered. Due to ongoing multi-organ deterioration, the patient was transferred to a center equipped for advanced surgical and circulatory support. An intra-aortic balloon pump was placed; however, the patient passed away despite the measures taken.

## Discussion

In older patients, AMI can be particularly challenging to diagnose and manage when mechanical complications arise, as this case demonstrates. Inferolateral AMI with RV involvement and dysfunction has a significant risk of hemodynamic instability. When present, if a VSD is not surgically repaired, it can lead to a high death rate and further complicate the clinical picture [9].

Recent studies have shown that the occurrence of VSD as a complication after AMI remains minimal in the current era, with patients treated using modern reperfusion methods reporting an incidence of roughly 0.2-0.3%. Prompt revascularization, which limits myocardial necrosis and the risk of septal rupture, is responsible for this significant reduction compared to the pre-reperfusion era. Despite being rare, VSD after AMI is still associated with considerable morbidity and mortality [10-12].

Prompt detection of mechanical complications such as VSD is vital. TTE plays a central role in evaluating ventricular function and identifying structural anomalies. However, instability often limits initial imaging, requiring ongoing reassessment, as demonstrated here [10,11,13,14].

This patient developed a VSD in the inferior portion of the interventricular septum as a consequence of an inferolateral myocardial infarction. This complication most likely resulted from extensive transmural necrosis in the territory supplied by the posterior descending artery. The inferior septum is particularly vulnerable to ischemia in inferolateral infarctions because its dual blood supply, from both the LAD and posterior descending arteries, can be compromised. Early intervention in myocardial infarction is essential to limit tissue damage and reduce the risk of mechanical complications such as VSD. When cardiogenic shock occurs, ongoing poor perfusion can worsen the injury and make septal rupture more likely. In addition, the composition of the thrombus and the extent of distal embolization may lead to microvascular obstruction and localized necrosis, creating conditions that favor the formation of septal defects. Understanding these processes helps clinicians stay alert and detect this potentially life-threatening complication as early as possible in similar patients. To restore blood flow and limit infarct extension, urgent revascularization of the blocked RCA was required. The slow flow and high thrombotic burden after PCI highlight the complexity and severity of this case. Hemodynamic support with norepinephrine and levosimendan was necessary to maintain adequate perfusion, while mechanical ventilation helped prevent respiratory collapse [7,15,16].

The consideration of the intra-aortic balloon pump highlights the necessity of improved circulatory support in cases of severe ventricular dysfunction. The only effective treatment for the VSD is still surgery. Percutaneous closure of VSD has emerged as an alternative to surgical treatment in selected centers due to the high morbidity and mortality risk associated with surgery. Given the patient's multi-organ dysfunction and deteriorating condition, transfer to a higher-level center was appropriate and necessary [17-19]. 

In patients with cardiogenic shock or severe hemodynamic instability, early surgery is sometimes unavoidable, even though the tissue is fragile and the operative risk is high. When possible, delaying repair for about one to two weeks allows the tissue to stabilize and significantly improves outcomes. In a cohort, patients who underwent surgery within the first week after infarction had a mortality of 40-50%. When surgery was delayed beyond seven days, mortality dropped to around 20-25%. These results are in line with the current European Society of Cardiology (ESC) guidelines, which recommend postponing surgery in stable patients whenever possible to allow the heart tissue to stabilize and improve the chances of survival [6,7].

Elderly patients who develop a VSD after an AMI, particularly when complicated by multi-organ failure, have a very poor prognosis. In one study, surgical repair was associated with a mortality of 33%, meaning roughly two out of three patients survived [6]. For patients who did not undergo surgery, the situation was much graver, with mortality approaching 94%. Even when procedures such as open surgical repair or percutaneous closure were performed, in-hospital mortality stayed high, over 40% [20]. These numbers show the severity of post-infarction VSD and emphasize the importance of early recognition and treatment. A multidisciplinary approach is required.

## Conclusions

The challenges of treating AMI in elderly patients are illustrated in this case study, particularly when it becomes aggravated by rare mechanical abnormalities like VSD. Early detection and intervention are necessary to improve the prognosis. TTE is still essential for detecting structural problems.

Stabilization required a combination of PCI, pharmacological hemodynamic support, and mechanical ventilation. However, definitive treatment of VSD and correction of hemodynamic compromise necessitate advanced circulatory support and surgical intervention.

In order to manage such difficult situations and provide individualized, coordinated care to achieve the best outcomes for older patients with severe AMI, an integrated, multidisciplinary strategy is important.
